# Disulfiram inhibits bacterial growth by inducing zinc-dependent reactive oxygen species

**DOI:** 10.3389/fmicb.2025.1619416

**Published:** 2025-07-17

**Authors:** Qinyu Luo, Zehua Wu, Yihang Pan, Yan Zhang

**Affiliations:** Department of Clinical Research Center, The Children’s Hospital, Zhejiang University School of Medicine, National Clinical Research Center for Child Health, Hangzhou, China

**Keywords:** *Escherichia coli*, colistin, zinc, ROS, disulfiram

## Abstract

**Introduction:**

The discovery of novel antimicrobial mechanisms among existing clinical drugs is urgently needed. Disulfiram, an FDA-approved treatment for alcohol dependence, exhibits broad-spectrum antibacterial effects. However, its mechanism of action remains incompletely understood.

**Methods:**

The antimicrobial activity of disulfiram was assessed using bacterial growth curves and colony-forming unit assays. Cytotoxicity was evaluated via propidium iodide staining and flow cytometry. Synergy with polymyxins or kanamycin was examined using checkerboard assays. RNA-seq was performed on disulfiram-treated E. coli, and differentially expressed genes were analyzed using the R package limma. Intracellular reactive oxygen species (ROS) levels were measured with fluorescent probes and flow cytometry.

**Results:**

Disulfiram exhibited bacteriostatic, but not bactericidal, effects against E. coli and S. aureus. However, it significantly enhanced the bactericidal activity of colistin or kanamycin, both in vitro and in a murine E. coli infection model. Transcriptomic analysis revealed oxidative stress and zinc-related responses in disulfiram-treated E. coli. The bacteriostatic effects were reversed by the ROS scavenger N-acetyl-l-cysteine and zinc chelators, whereas zinc supplementation enhanced ROS production and growth inhibition.

**Discussion:**

This study identifies a zinc-dependent ROS-mediated mechanism underlying the bacteriostatic activity of disulfiram. Although the in vivo concentrations of disulfiram during standard therapy are below its MIC, its synergistic effect with colistin suggests clinical relevance as an adjuvant. Disulfiram-induced redox stress and zinc modulation likely compromise bacterial antioxidant defenses and membrane integrity. These findings support further investigation of dithiocarbamate-based compounds as potential adjuvants or scaffolds for novel antimicrobial development.

## 1 Introduction

Bacterial diseases cause significant mortality globally and place a major burden on public health systems (GBD 2019 Antimicrobial Resistance Collaborators, 2022). Bacterial infections cause most cases of sepsis, an extreme host reaction to an infection. The number of sepsis-related deaths reached 11 million in 2017, resulting in great global health destruction (Darby et al., 2023; Bush and Bradford, 2020). The emergence of multidrug-resistant bacteria poses a further threat to existing antimicrobial treatment regimens. Currently, last-resort antibiotics, including colistin, are losing effectiveness due to the spread of resistance genes among bacteria (Darby et al., 2023; Bush and Bradford, 2020). Hence, the discovery of new antibiotics and the exploration of additional antimicrobial mechanisms of medicines currently used in the clinic are urgently needed.

Disulfiram has been the first FDA-approved drug for alcohol use disorder since 1951 (Suh et al., 2006). However, this chemical compound is so versatile that more than half a century later, studies continue to reveal additional medical uses. For example, disulfiram has antitumor effects on many types of human cancer (Lu et al., 2021); it inhibits neutrophil extracellular traps to limit SARS-CoV-2 lung injury (Adrover et al., 2022); and it normalizes the weight of obese mice (Bernier et al., 2020). Among these applications, one of the most attractive functions of disulfiram is to affect the growth of a broad spectrum of bacteria, against both Gram-negative pathogens (e.g., *F. tularensis*) and Gram-positive species (e.g., *S. aureus*) (Chen et al., 2023; Chen et al., 2020; Hamblin et al., 2019; Thakare et al., 2019) and even Mycoplasma (Meneguello et al., 2022). This function endows disulfiram with potential for the treatment of bacterial infections.

Nevertheless, the universal mechanisms underlying the antibacterial effects of disulfiram remain elusive. Studies have shown that disulfiram can inhibit aldehyde dehydrogenase-like proteins in *F. tularensis* or metallo-β-lactamases (MBLs) in Enterobacterales (Chen et al., 2020; Hamblin et al., 2019). However, some bacteria that disulfiram inhibits do not possess such proteins. Disulfiram forms a complex with copper and exerts synergistic killing effects on *M. tuberculosis* (Dalecki et al., 2015). However, this synergistic effect is observed primarily with mycobacteria. Also, the antibacterial activity of disulfiram may partly attribute to its electrophilic nature and ability to form disulfide bonds with thiol-containing bacterial proteins, thereby disrupting essential enzyme function. Nonetheless, the full mechanism and its therapeutic potential still warrant further investigation.

This study aimed to quantify the effect of disulfiram against bacteria and elucidate the possible mechanisms of disulfiram through transcriptomic and phenotypic assays. Through RNA sequencing and the use of reactive oxygen species (ROS) inhibitors, we exploited an additional mechanism by which disulfiram inhibits bacteria. We found that disulfiram halted bacterial growth by increasing zinc-dependent intracellular ROS levels, and ROS scavengers or zinc chelators could restore bacterial growth. These findings provide a potential therapeutic strategy for treating bacterial infectious diseases.

## 2 Materials and methods

### 2.1 Animals and ethics approval

C57BL/6 wild-type (WT) mice aged 6–8 weeks were purchased from Shanghai SLAC Laboratory Animal Cooperation. All the mice were housed in a specific pathogen-free and temperature-controlled standard environment in accordance with the National Institutes of Health Guide for Care and Use of Laboratory Animals. The animal experimental protocols were approved by the Animal Review Committee of Zhejiang University School of Medicine and were in compliance with institutional guidelines.

### 2.2 Chemical compounds

The complete information for all antibiotics and compounds used in this study was listed as followed: disulfiram (MedChemExpress, HY-B0240, in DMSO), tetracyclin (Aladdin, T105493-10 g, in DMSO), colistin (Solarbio, C9810, in DMSO), meropenem (Aladdin, M427157-1 mL, in DMSO), ampicillin (Aladdin, A105483-5 g, in DMSO), sulbactam (Aladdin, E129319-1 g, in DMSO), N-Acetyl-L-cysteine (MedChemExpress, HY-B0215, in DMSO), ZnCl_2_ (MedChemExpress, HY-Y0420, in H_2_O), Tpen (Aladdin, N159625-250 mg, in DMSO), polymyxin B (Aladdin, P105490-1 g, in DMSO), kanamycin (Aladdin, K331597-200 mg, in DMSO), miconazole (Macklin, M909105-25 g, in DMSO), benzoquinone (Macklin, B802580-5 g, in DMSO), CuGlu (Aladdin, D133471-25 g, in H_2_O), TTM (Aladdin, A189030-200 mg, in DMSO), Ferric (III) Citrite (MedChemExpress, HY-B1645, in H_2_O), deferoxamine (MedChemExpress, HY-B0988, in DMSO), Dimethyl sulfoxide (MedChemExpress, HY-Y0320), coin oil (MedChemExpress, HY-Y1888).

### 2.3 Bacterial strains

The laboratory strains used in this study were obtained from the American Type Culture Collection (ATCC) and included *Escherichia coli* (ATCC 25922), *Staphylococcus aureus* (ATCC 25923), methicillin-resistant *Staphylococcus aureus* (MRSA; ATCC 43300), *Klebsiella pneumoniae* (ATCC 13883), *Pseudomonas aeruginosa* (ATCC 27853), and *Acinetobacter baumannii* (ATCC 17978). Clinical strains (*E. coli* and *A. baumanii*) were collected from Microbiology Laboratory of Children’s hospital, 2023–2024.

### 2.4 Determination of minimum inhibitory concentration (MIC_50_)

The MIC_50_ values of antibiotics were determined using the broth microdilution method according to CLSI guidelines. Bacterial suspensions (0.5 McFarland standard) were diluted 1:100 in cation-adjusted Mueller-Hinton broth and dispensed into 96-well plates containing two-fold serial dilutions of antibiotics (0.25–256 μg/mL). Plates were incubated at 37°C for 18–22 h. MIC_50_ was defined as the lowest concentration of antibiotic that inhibited ≥50% of bacterial growth, assessed by optical density at 600 nm (OD600). Experiments were performed in triplicate.

### 2.5 Bacterial killing analysis

Bacteria were cultured to log phase and diluted to an OD600 of 0.2–0.3. Disulfiram and colistin were added as indicated. The bacterial cells were subsequently washed 4 h later. The cell numbers were determined by dilution, incubation on Luria–Bertani agar plates at 37°C overnight, and calculation by multiplying the countable bacterial colony units by the number of dilutions.

### 2.6 Bacterial growth curve

Bacteria were cultured to log phase, diluted to 5 × 10^5^ colony-forming units (CFUs)/ml in 200 μL of LB and placed in a 96-well microliter plate. The drugs and compounds were added as indicated. The growth curve of bacteria was monitored by measuring the absorbance at 600 nm using a microplate reader (SpectraMax190, Molecular Devices) over a 24 h period.

### 2.7 Checkerboard assay

Drug synergism was tested in checkerboard assays as described (Chen et al., 2023). Briefly, disulfiram and other antibiotics or compounds were twofold serially diluted in an 8 × 8 matrix within a 96-well microliter plate. Bacteria were grown to log phase and diluted in each well to 5 × 10^5^ CFUs/ml in 200 μL of MHB. After 18–22 h of incubation at 37°C, the absorbance of each well at 600 nm was measured with a microplate reader (SpectraMax190, Molecular Devices). The fractional inhibitory concentration index (FICI) was calculated accordingly, and a value less than 0.5 demonstrated synergy.

### 2.8 Membrane permeability assay

The membrane permeability rate of the cells was determined by propidium iodide (PI, 2.5 μg/mL) staining and flow cytometry. A minimum of 20,000 events per sample were acquired using the PE fluorescence channel during flow cytometry analysis. The analysis was performed using a DxFLEX or a CytoFLEX LX (Beckman Coulter, United States).

### 2.9 *In vivo* model

We used an *E. coli* murine intra-abdominal infection model to analyze the synergistic effect of disulfiram and colistin *in vivo*. Briefly, *E. coli* bacteria were seeded on Luria–Bertani (LB) agar plates and cultured overnight. A single clone was selected for culture in liquid LB medium and incubated with shaking at 200 rpm at 37°C for another 12 h. The bacterial suspension was then prepared at a concentration of 7 × 10^6^ colony-forming units (CFUs) per 100 μL of PBS. To induce intra-abdominal infection, the mice were intraperitoneally administered 100 μL of live *E. coli* suspension. Thirty minutes later, disulfiram (resolved in corn oil), colistin (resolved in corn oil) or control solvent (corn oil) was administered to the mice intraperitoneally as indicated. Mortality was assessed hourly for 72 h.

### 2.10 Microscopy

Bacteria were cultured to log phase and diluted to an OD600 of 0.2–0.3. Disulfiram and other antibiotics were added as indicated. Four hours later, the cells were inoculated on a slide and subjected to microscopic observation. Images were taken with an Olympus IX73 inverted microscope, and the cell lengths were measured using ImageJ software.

### 2.11 Motility assay

Motility assay was tested as described (Ejim et al., 2011). Briefly, swimming motility was observed on 0.3% (w/v) agar medium composed of 10 g/L trypticase peptone, 10 g/L NaCl and 5 g/L yeast extract. The bacterial cells were cultured to log phase, standardized to 2–3 × 10^6^ CFUs/ml, point inoculated onto a swim plate and incubated for 20 h at 37°C. The swimming areas were calculated using ImageJ software.

### 2.12 RNA sequencing and differential expressed genes analysis

*E. coli* were grown to log phase and cultured with disulfiram or the solvent DMSO (*n* = 3 biologically independent samples) for 2 h. The cells were collected, total RNA was extracted using TRIzol^®^ Reagent according to the manufacturer’s instructions (Invitrogen), and the genomic DNA was removed using DNase I (TaKara). The RNA was then quantified with an ND-2000 (NanoDrop Technologies) and subjected to library construction, sequencing and data analysis. The RNA-seq strand-specific libraries were prepared with a TruSeq RNA sample preparation kit from Illumina (San Diego, CA) using 5 μg of total RNA. In brief, rRNA was removed using a RiboZero rRNA removal kit (Epicenter) and fragmented in fragmentation buffer. cDNA synthesis, end repair, A-base addition and ligation of the Illumina-indexed adaptors were performed according to Illumina’s protocol. Paired-end libraries were sequenced on an Illumina NovaSeq 6000 (Shanghai BIOZERON Co., Ltd.). The raw paired-end reads were trimmed and quality controlled in Trimmomatic with the following parameters: SLIDINGWINDOW: 4:15 MINLEN:75 (version 0.36; http://www.usadellab.org/cms/uploads/supplementary/Trimmomatic). Then, the clean reads were separately aligned to the reference genome in orientation mode using Rockhopper (http://cs.wellesley.edu/~btjaden/Rockhopper/) software. The differentially expressed mRNAs with a fold change > 2 or < 0.5 and *p* value < 0.05 were selected via the R package edgeR (https://bioconductor.org/packages/release/bioc/html/edgeR.html).

### 2.13 Clusters of transcriptomic changes

The transcriptomes from each chemical compound-treated *E. coli* experiment were downloaded from NCBI (colistin-treated *E. coli*: GSE220559; kanamycin-, H_2_O_2_-treated *E. coli*: GSE56133). Differentially expressed genes were analyzed using the bioconductor package limma (https://www.bioconductor.org/packages/release/bioc/html/limma.html). The clustering of transcriptomic changes was performed using the R package pheatmap (https://cran.r-project.org/web/packages/pheatmap/index.html) with the clustering method “ward. D.”

### 2.14 ROS measurement

Cellular ROS levels were measured using CellROX (Thermo Fisher Scientific, United States, C10444) probes. Briefly, bacteria were cultured to log phase, diluted to an OD600 of 0.2–0.3, cultured with the indicated compounds for 1 h, and then, the probes were loaded at 5 μM at 37°C for 30 min. The cells were washed twice and resuspended in PBS, and then flow cytometry was performed using a DxFLEX or a CytoFLEX LX (Beckman Coulter, United States). A minimum of 20,000 events per sample were acquired using the FITC fluorescence channel during flow cytometry analysis.

### 2.15 Data analysis

The data are shown as the means ± SDs. Differences were analyzed by Student’s *t* test or one-way ANOVA with Tukey’s *post hoc* analysis. Kaplan–Meier survival analysis was performed for survival experiments. A *p* value < 0.05 was considered statistically significant. Statistical analysis was carried out in GraphPad Prism 9.3 (GraphPad Software).

## 3 Results

### 3.1 Evaluation of disulfiram’s bacteriostatic activity

**Figure 1:**
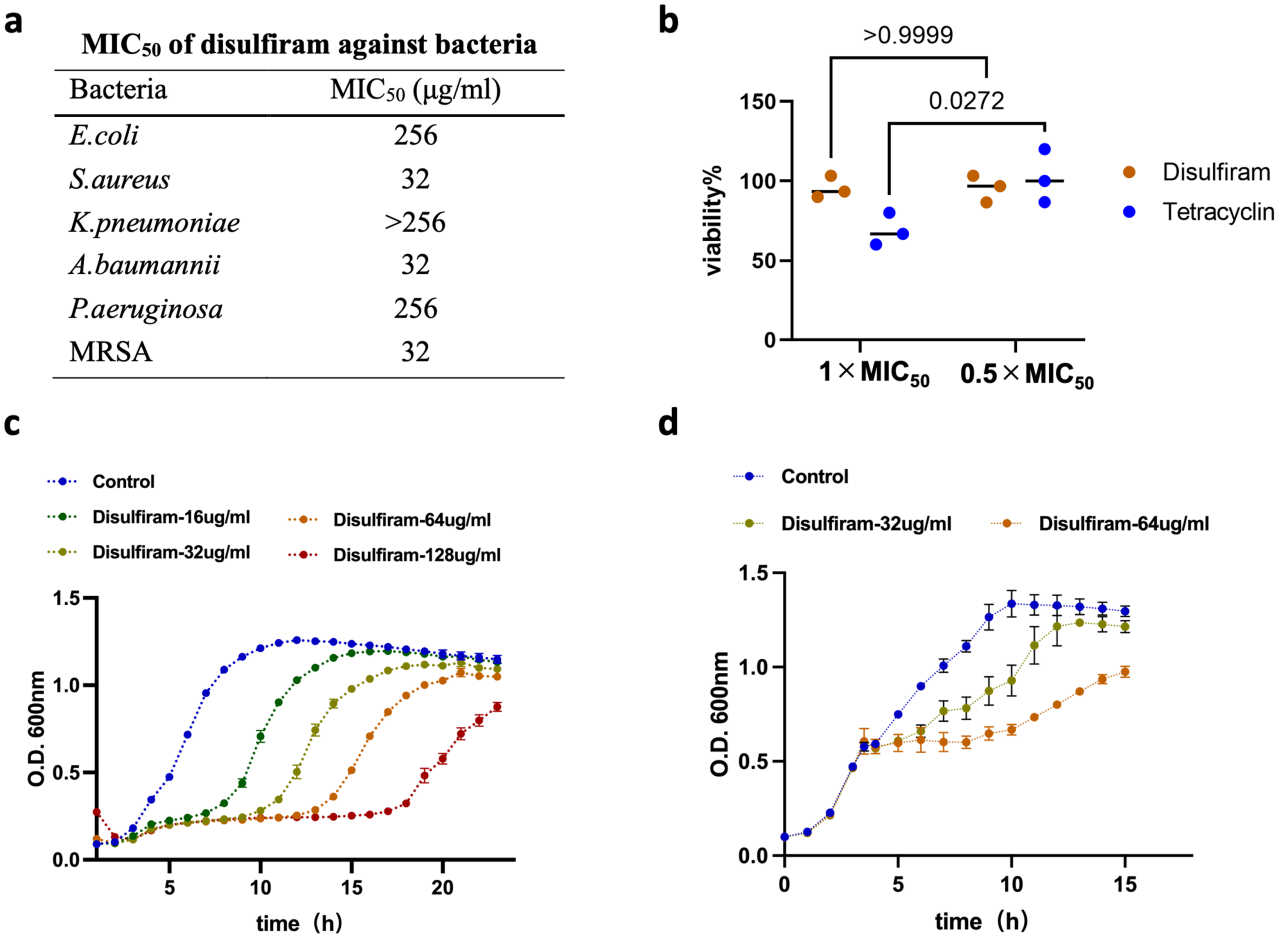
Evaluation of disulfiram’s bacteriostatic activity. **(a)** MIC_50_ values of disulfiram against multiple pathogens, including *E. coli*, *S. aureus*, *K. pneumoniae*, *A. baumannii*, *P. aeruginosa* and methicillin-resistant *S. aureus* (MRSA). **(b)** Viability of *E. coli* in the presence of tetracycline (MIC_50_: 0.5 μg/mL) or disulfiram (MIC_50_: 256 μg/mL). **(c)** Growth curve of *E. coli* treated with disulfiram at the indicated concentrations at the beginning of the experiment. **(d)** Growth curve of *E. coli* treated with disulfiram at the indicated concentration for 3.5 h. Data in c and d are the means ± SD of three biological replicates. *p* values were determined using unpaired, two-tailed Student’s *t* tests.

To understand the effects of disulfiram on bacteria, we first monitored the 50% minimal inhibitory concentration (MIC_50_) of disulfiram on the 6 common clinical bacteria including *E. coli*, *S. aureus*, *K. pneumoniae*, *A. baumannii*, *P. aeruginosa* and methicillin-resistant *S. aureus* (MRSA). The results revealed that disulfiram had relatively high MIC_50_ values against these bacteria, with values of 256 μg/mL against *E. coli* and *P. aeruginosa* and 32 μg/mL against *S. aureus*, *A. baumannii* and MRSA (Figure 1a). These concentrations are higher than typical clinical plasma levels, suggesting *in vitro* activity may not fully translate *in vivo*. Next, we tested whether disulfiram killed bacteria at high concentrations. We used tetracycline, a bacteriostatic drug (Maier et al., 2021), as a control. The results revealed that while tetracycline killed *E. coli* at 1 × MIC_50_ (0.5 μg/mL), disulfiram had nearly no killing effect on *E. coli* at 1 × MIC_50_ (256 μg/mL; *n* = 3, 95.53 ± 6.93% vs. 68.90 ± 10.18%, *p* = 0.0389), indicating that disulfiram had no bactericidal effect (Figure 1b). A microplate reader was then used to monitor the bacterial growth curve to further observe the effect of disulfiram on the growth of *E. coli*. Disulfiram delayed the exponential growth phase of *E. coli* in a concentration-dependent manner (Figure 1c). Moreover, disulfiram halted *E. coli* growth instantly at the exponential phase (Figure 1d), indicating that disulfiram has a fast bacteriostatic effect on *E. coli*. These results demonstrated that disulfiram is a fast bacteriostatic drug with no bactericidal effect.

### 3.2 Combination effects of disulfiram and colistin on bacterial survival

**Figure 2:**
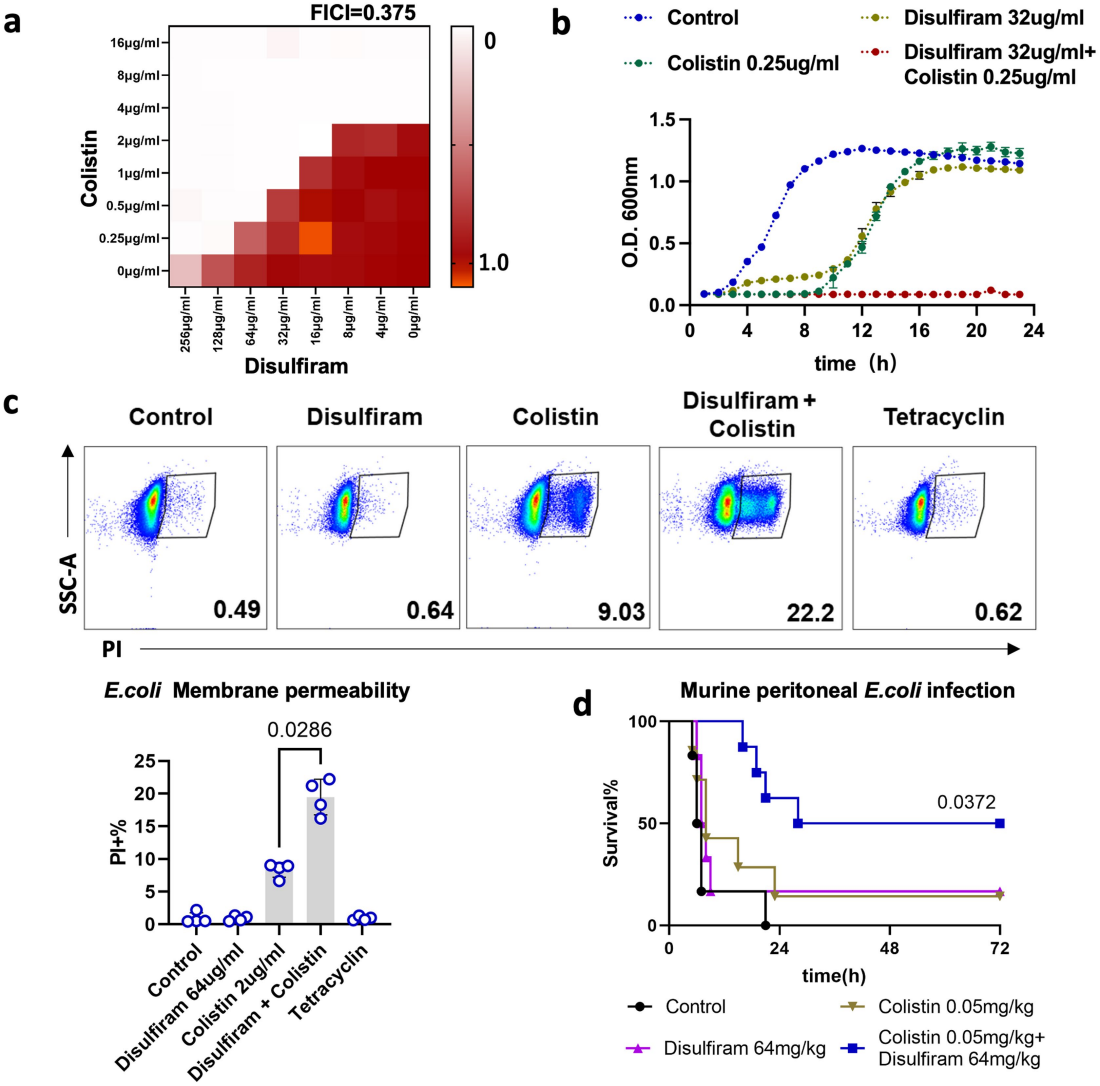
Combination effects of disulfiram and colistin on bacterial survival. **(a)** Checkerboard assay of disulfiram and colistin against *E. coli*. The optical density (OD) at 600 nm was measured after 20 h of incubation at 37°C. The data are expressed as the absorbance at 600 nm and represent biological replicates. **(b)** Growth curves of *E. coli* in the presence of disufiram and colistin for 24 h. The means ± SD of three biological replicates are shown. **(c)** Membrane permeability assay of *E. coli* treated with disulfiram (64 μg/mL), colistin (2 μg/mL) or tetracycline (0.5 μg/mL). *p* values were determined using the Mann–Whitney U test. **(d)** Survival rates of mice in the peritonitis–sepsis model (*n* = 6 ~ 8) over 72 h after infection with a lethal dose of *E. coli* (3.0 × 10^7^ CFUs) treated with disulfiram or colistin alone or in combination. *p* values were determined using the Mantel–Cox test.

Antibiotic sensitizers or adjuvants play important roles in preventing microbial infections through the collaboration of antibiotics (Wang et al., 2024). Fast bacteriostatic drugs are often used as adjuvants for stationary-phase bactericidal drugs. A recent study showed that disulfiram could bind to New Delhi metallo-β-lactamase 1 (NDM-1) and resensitize drug-resistant bacteria to colistin and meropenem (Chen et al., 2023). However, we found that 32 μg/mL disulfiram could reduce the MIC of colistin on *E. coli* by 4-fold, which did not involve antibiotic resistance genes (FICI = 0.375, Figure 2a). The combination of disulfiram and colistin amplified the bactericidal effect of colistin alone (Figure 2b; Supplementary Figure S2a). Similarly, disulfiram also synergized with polymyxin B (FICI = 0.25) or kanamycin (FICI = 0.375) against *E. coli* or with colistin (FICI = 0.375) or kanamycin (FICI = 0.5) against *A. baumannii* (Supplementary Figure S1). The clinical isolates that generally cause sepsis or Healthcare-Associated Infections (HAIs) may have different sensitivity patterns than lab strains. We have also included clinical isolated strains for assay. The result showed that disulfiram synergized with colistin against clinical isolated *E. coli* (FICI = 0.047) and *A. baumannii* (FICI = 0.125, Supplementary Figure S1). A cell permeability assay revealed that although disulfiram alone did not permeabilize the cell membrane, it augmented the cell membrane damage caused by colistin (*n* = 4, 19.48 ± 2.74% vs. 8.31 ± 1.13%, *p* = 0.0286, Figure 2c). Moreover, we applied disulfiram to an animal model of *E. coli* intraperitoneal infection and found that combination therapy with disulfiram and colistin resulted in better outcomes than treatment with colistin alone for the disease (*n* = 7–8, 70.83 ± 20.41 h vs. 51.02 ± 34.85 h, *p* = 0.013, Figure 2d; Supplementary Figure S2b). These results indicated that disulfiram could act as a potential antibiotic sensitizer or adjuvant with stationary-phase bactericidal antibiotics, including colistin, polymyxin B or kanamycin.

### 3.3 Analysis of bacterial motility and morphology under disulfiram treatment

**Figure 3:**
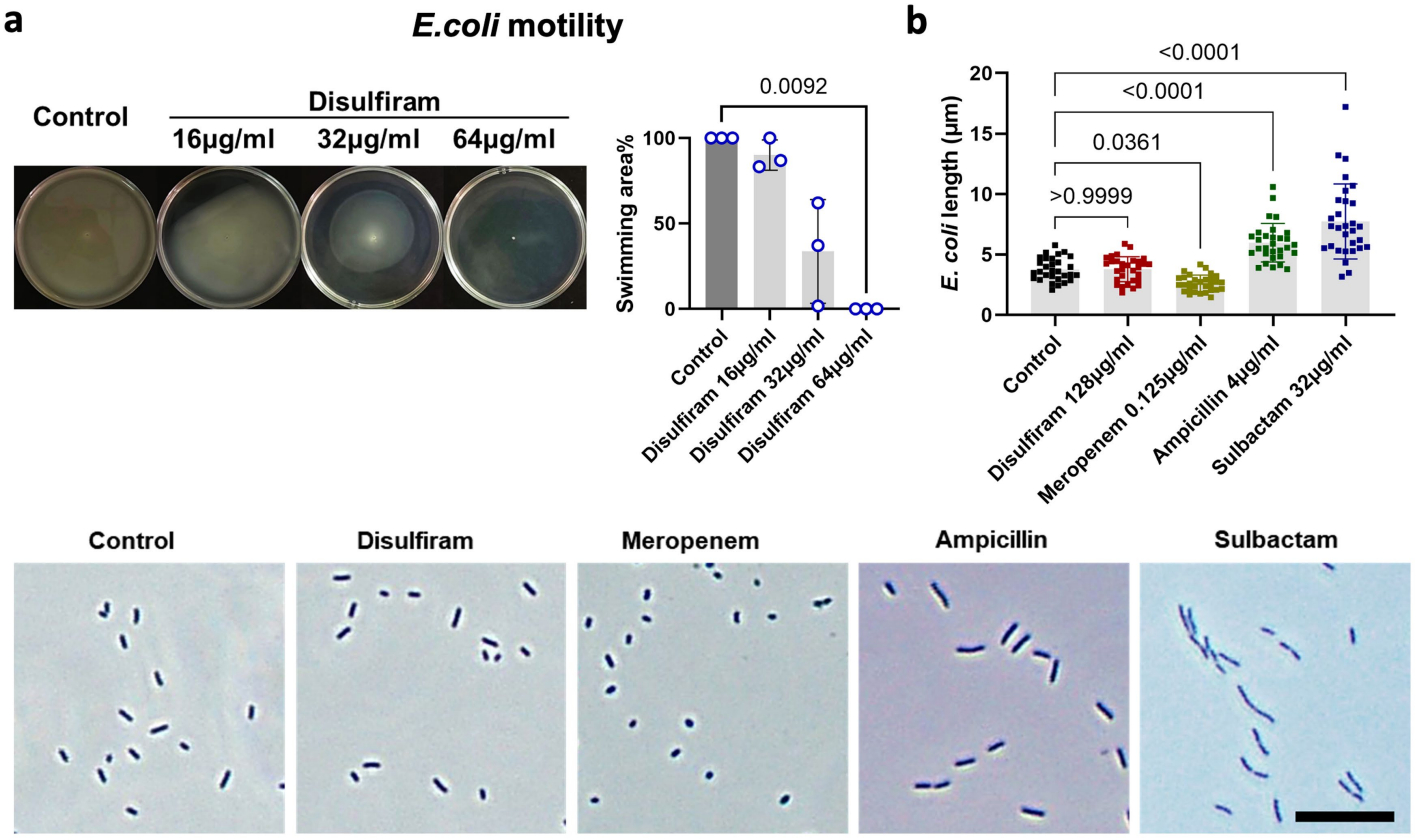
Analysis of bacterial motility and morphology under disulfiram treatment. **(a)** Swimming assay of *E. coli* treated with disulfiram at the indicated concentrations. Images represent biological replicates. **(b)** Microscopy images and lengths of *E. coli* treated with disulfiram, meropenem, ampicillin or sulbactam (0.5 × MIC_50_). Images represent biological replicates. *p* values were determined using one-way ANOVA.

Next, we sought to investigate the effect of disulfiram on *E. coli*. Swimming motility is vital to bacteria, as this process supports their movement toward resources. We found that under disulfiram treatment, the motility of *E. coli* was inhibited (At 64 μg/mL, *n* = 3, 100 ± 0% vs. 0 ± 0%, *p* = 0.0092, Figure 3a). This may result from decreased swimming ability or potential viability loss. Studies have shown that motility patterns are associated with bacterial cell shape and that the antibacterial cellular pathways of chemical compounds are associated with bacterial cytology profiles (Nonejuie et al., 2013; Wadhwa and Berg, 2022). Under microscopic observation, while sulbactam, ampicillin and meropenem, which are penicillin-binding protein (PBP)-targeting antibiotics, changed the length of *E. coli*, disulfiram did not have an effect (*n* = 30, 3.78 ± 1.04 μm vs. 3.77 ± 0.94 μm, *p* > 0.9999, Figure 3b), indicating that the target of disulfiram may not be bacterial PBPs (Penwell et al., 2015).

### 3.4 Transcriptomic profiling of *Escherichia coli* in response to disulfiram exposure

**Figure 4:**
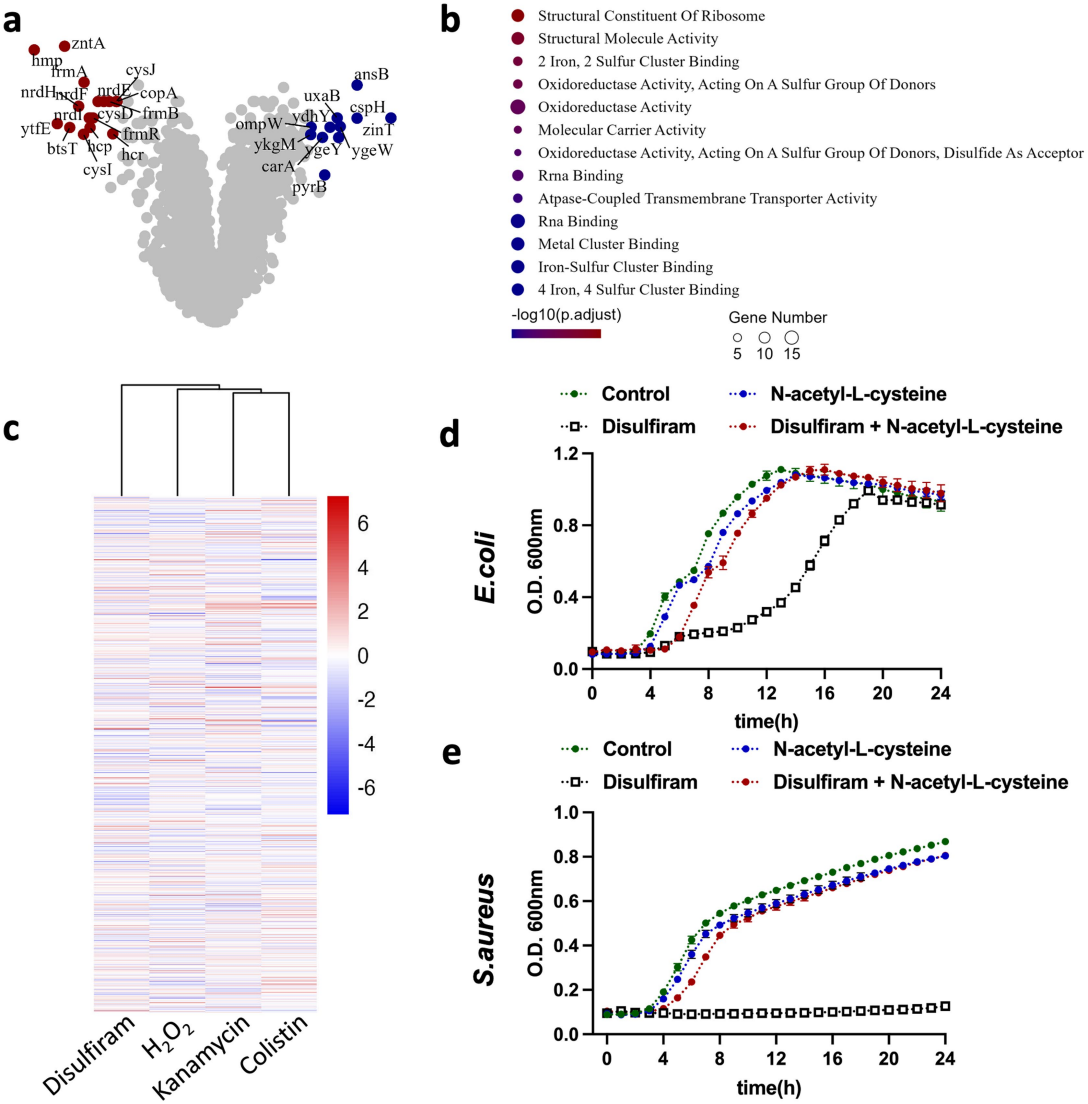
Transcriptomic profiling of *E. coli* in response to disulfiram exposure. **(a)** Transcriptome analysis of *E. coli* treated with disulfiram (64 μg/mL) for 4 h. Genes with increases or decreases of log [fold change] > 4 and *p* values < 0.01 are shown in the original list of 4,798 genes (*n* = 3 biological replicates). **(b)** Molecular function changes in differentially expressed genes in disulfiram (64 μg/mL, 4 h)-treated *E. coli*. Genes with increases or decreases of log [fold change] > 2 and *p* values < 0.05 were included and analyzed (*n* = 3 biological replicates). **(c)** Transcriptome profiles of *E. coli* treated with kanamycin, H_2_O_2_, disulfiram or colistin were clustered as described in the methods. A total of 3,218 genes were annotated universally across samples and analyzed. **(d)** Growth curves of *E. coli* in the presence of disufiram (32 μg/mL) and N-acetyl-L-cysteine (5 mM) for 24 h. **(e)** Growth curves of *S. aureus* in the presence of disufiram (32 μg/mL) and N-acetyl-L-cysteine (5 mM) for 24 h. Data in **(d,e)** are the means ± SD of three biological replicates.

To study the detailed mechanisms by which disulfiram inhibits bacterial growth in detail, we subjected disulfiram-treated *E. coli* to RNA sequencing (RNA-seq) to study the transcriptome changes. Compared with the control *E. coli* group, disulfiram-treated *E. coli* expressed higher levels of antioxidant-related genes, including hmpA, cysI, nrdH, and frmA; higher levels of the iron–sulfur cluster repair-related gene ytfE; lower levels of zinT and higher levels of zntA, two genes associated with zinc binding and zinc translocating; higher levels of the pyruvate uptake-related gene btsT; and lower levels of the nucleotide synthesis-related gene ygeW (Figure 4a). The top genes among the differentially expressed genes (DEGs) were hmpA, which encodes flavohemoglobin and is linked to the cell response to oxidative or nitrosative stress (Bonamore and Boffi, 2008). Azoles and quinones are reported to be inhibitors and substrates of flavohemoglobin (Moussaoui et al., 2018; Nobre et al., 2010). We first tested whether these compounds could augment the effect of disulfiram. The results of the checkerboard assay suggested that inhibiting or monitoring flavohemoglobin only had a very mild effect on disulfiram inhibition of *E. coli* (Supplementary Figures S2c,d), indicating that drugs or chemicals that target flavohemoglobin cannot efficiently enhance the effect of disulfiram. Pathway enrichment analysis further revealed that the differentially regulated genes were enriched in the iron-sulfer cluster binding, oxidoreductase activity and RNA binding pathways (Figure 4b). These results indicate that disulfiram may affect the growth of *E. coli* through oxidant activity, leading to iron-sulfer cluster damage and the suspension of genetic material synthesis. To provide a preliminary overview of potential similarities in gene expression profiles, we performed clustering analysis by comparing our transcriptomic data with publicly available datasets that examined RNA expression changes in *E. coli* in response to various bacteriostatic and bactericidal agents. The results revealed that the disulfiram-induced transcriptomic changes were closely associated with those caused by H_2_O_2_, which cause oxidative damage to pathogens (Schairer et al., 2012). In contrast, bactericidal drugs, including kanamycin or colistin, had relatively distinct transcriptomic changes (Figure 4c). Furthermore, we verified that the ROS inhibitor N-acetyl-L-cysteine (NAC) fully prevented the bacteriostatic effect of disulfiram on both *E. coli* and *S. aureus* (Figures 4d,e). These results confirmed that disulfiram inhibits bacterial growth by inducing ROS.

### 3.5 Mechanistic investigation of disulfiram-mediated growth inhibition

The coeffect of metal ions is proposed as a biological mechanism of disulfiram (Skrott et al., 2017; Gao et al., 2022). Indeed, disulfiram and copper kill *Mycobacterium tuberculosis* in a synergetic manner (Dalecki et al., 2015). However, when copper gluconate was used as a Cu^2+^ supply in *E. coli* growth medium, we found the opposite effect that copper ions counteracted the inhibitory effect of disulfiram on *E. coli* growth (Supplementary Figure S3a). We proposed that copper ions and the metabolites of disulfiram (ditiocarb and diethyldithiocarbamate, which have bacteriostatic effects similar to those of disulfiram) could form chemical complexes (bis (diethyldithiocarbamate)–copper), which may cover the effective bacteriostatic group of disulfiram. Moreover, the copper chelator tetrathiomolybdate (TTM) had a very weak synergistic effect with disulfiram on the growth of *E. coli* (Supplementary Figure S3a). These results indicated that the disulfiram-mediated inhibition of bacterial growth was independent of copper ions.

As shown by the transcriptomic results, Fe-S cluster damage and repair participate in the mechanism of disulfiram. Ferric ions may induce ROS through the Fenton reaction. However, upon cotreatment with disulfiram, the ferric chelator DFO (deferoxamine) only weakly inhibited the growth of *E. coli*, whereas ferric citrate had no effect (Supplementary Figure S3b).

**Figure 5:**
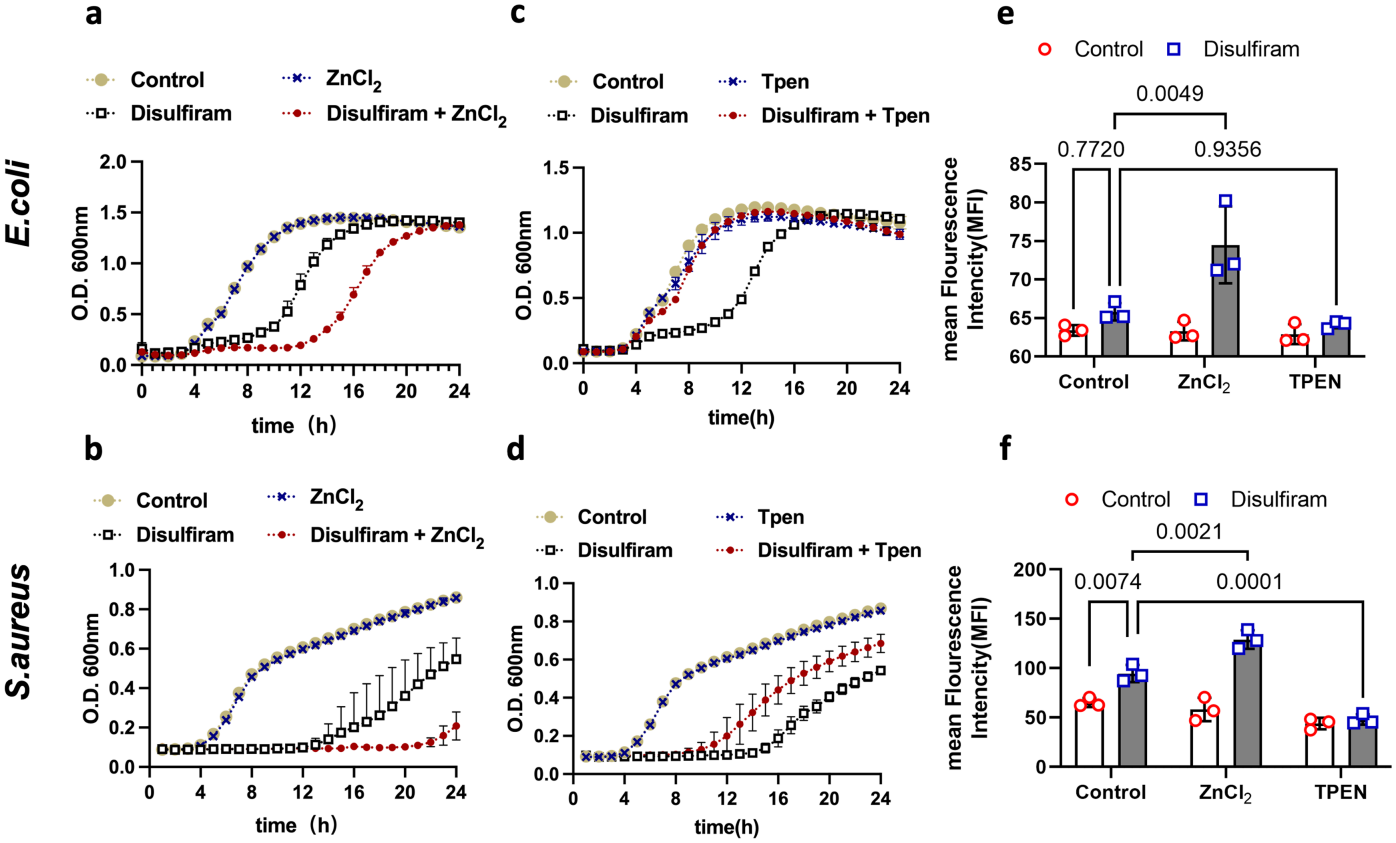
Mechanistic investigation of disulfiram-mediated growth inhibition. **(a,c)** Growth curves of *E. coli* treated with disulfiram (32 μg/mL), ZnCl_2_ (10 μM), or TPEN (10 μM) or their combination as indicated for 24 h. **(b,d)** Growth curves of *S. aureus* treated with disulfiram (32 μg/mL), ZnCl_2_ (10 μM), TPEN (10 μM) or their combination as indicated for 24 h. **(e)** ROS levels of *E. coli* treated with disulfiram (32 μg/mL), ZnCl_2_ (10 μM), TPEN (10 μM) or their combination as indicated. **(f)** ROS levels of *S. aureus* treated with disulfiram (32 μg/mL), ZnCl_2_ (10 μM), TPEN (10 μM) or their combination as indicated. The data in **(a,d)** are presented as the means ± SDs, and *p* values were determined using ordinary two-way ANOVA. The data in **(a–d)** are the means ± SD of three biological replicates.

Zinc binding and zinc translocation may play a role in this process, as the zinT and zntA genes were upregulated in disulfiram-treated *E. coli*. Surprisingly, we found that while zinc ions augmented the inhibitory effect of disulfiram on both *E. coli* (Figure 5a) and *S. aureus* (Figure 5b), the zinc chelator TPEN (N, N, N′, N′-tetrakis- (2-pyridylmethyl) ethylenediamine) counteracted the inhibitory effect of disulfiram, fully restored the growth of *E. coli* (Figure 5c) and partly restored the growth of *S. aureus* (Figure 5d). These results suggested that zinc ions play important roles in the bacteriostatic mechanisms of disulfiram. Therefore, disulfiram has a ROS-dependent bacteriostatic effect. We next tested the relationship between ROS and zinc. We loaded CellROX, a fluorescent ROS probe, into bacteria as an indicator of ROS and found that while zinc ions augmented the disulfiram-induced increase in ROS level in *E. coli* (*n* = 3, 74.47 ± 4.98 vs. 65.80 ± 1.13, *p* = 0.0049) and *S. aureus* (*n* = 3, 129.0 ± 9.54% vs. 94.53 ± 8.63%, *p* = 0.0021), TPEN treatment fully suppressed this increase in both *E. coli* (*n* = 3, 64.13 ± 0.47% vs. 65.80 ± 1.13%, *p* = 0.9356) and *S. aureus* (*n* = 3, 47.97 ± 5.24% vs. 94.53 ± 8.63%, *p* = 0.0001, Figures 5d,e). These results suggested that the bacteriostatic effect of disulfiram is mediated by zinc ions, and that ROS levels depend on zinc.

## 4 Discussion

Our findings confirmed that disulfiram halted the growth of bacteria by inducing zinc-dependent ROS in bacteria, providing further mechanisms for the existing bacteriostatic drugs. While previous studies have reported the antibacterial effects of disulfiram and its role as a metallo-β-lactamase inhibitor or antibiotic adjuvant, our results are the first to identify zinc-dependent ROS generation as a key mechanism underlying its bacteriostatic activity.”

Even with maximal FDA-approved dosing (500 mg/day), disulfiram is only on the order of a few μg/mL in the plasma and this is lower than the reported antibacterial MIC₅₀ (~256 μg/mL). Thus, plasma disulfiram concentrations under typical therapy are too low to reach the concentrations required for direct antimicrobial activity. We speculated that an adjuvant effects of disulfiram at lower concentrations may be more clinically relevant than direct bacteriostatic activity. Therefore, future studies should evaluate whether the observed adjuvant effects at lower, clinically relevant concentrations may offer therapeutic benefit in combination with existing antibiotics. Disulfiram works in synergism with colistin. Disulfiram is reported to inhibit mRNA expression of mcr-1 and bind to MCR enzymes, thereby amplify membrane damage ability of colistin (Chen et al., 2023). However, in our study, the colistin resistance gene is not present or expressed in the *E. coli* 25,922 genome. This indicated that disulfiram may strengthen the ability of colistin by some other universal mechanisms. On one hand, disulfiram may weaken the outer membrane of bacteria via metal chelation, facilitating colistin’s penetration, on the other hand, transcriptomic analysis revealed disulfiram-induced oxidative stress pathways, which may amplify colistin’s bactericidal effect by overwhelming bacterial antioxidant defenses. This universal mechanism may potentially extend the usage of disulfiram in clinical circumstances. While disulfiram has demonstrated antibacterial activity *in vitro*, the *in vivo* mechanism remains to be fully elucidated. Our *in vitro* data of disulfiram suggested a substantial contribution of direct antibacterial action. However, additional immunological profiling will be needed to assess potential immune-mediated effects.

Bacteriostatic drugs are important in the treatment of multiple infectious diseases. For example, tigecycline, the next generation of tetracycline, is regarded as a last resort for multidrug-resistant (MDR) bacterial infection (Fang et al., 2020); linezolid is an important alternative in the treatment of vancomycin-resistant enterococci (VAE) or MRSA/MSSA (Bender et al., 2018; Kawasuji et al., 2023). However, these final-line antibiotics are now facing serious levels of resistance from bacteria. Currently available bacteriostatic drugs, including folate inhibitors (sulfonamides and trimethoprim), tetracyclines, and macrolides, either inhibit bacterial growth via folate synthesis inhibition or target ribosomal elements to affect protein synthesis (Sachin and Parag, 2021). Hydrogen peroxide, which generates ROS to kill bacteria, actually kills bacteria at a very high concentration (i.e., half of bacteria can be killed in 10 mM (0.03%) at 1 h) (Thomas et al., 1994). Studies have also reported that ROS do not participate in the bactericidal effects of multiple antibiotics (Keren et al., 2013). However, ROS can contribute to antibiotic lethality (Dwyer et al., 2014), which suggests that ROS inducers could be potentially important adjuvants for current bactericidal antibiotics. In our study, we found that disulfiram induced bacterial ROS, inhibited bacterial growth and augmented the effect of colistin *in vitro* and *in vivo*. These findings suggested that disulfiram or other dithiocarboxy acid-containing chemicals might be potential adjuvants with additional antimicrobial mechanisms.

H_2_O_2_, paraquat and NO are chemicals related to oxidative damage. H_2_O_2_ directly generates ROS; paraquat is a well-known ROS inducer; and NO causes oxidative stress through reactive nitrogen species (RNS) (Jomova et al., 2023). Disulfiram treatment of *E. coli* induced the expression of multiple redox detoxification genes, indicating that the redox cycle of bacteria is affected. However, how disulfiram induces ROS remains elusive, as dithiocarboxy acid tends to alter the redox cycle, and disulfide bonds alone do not affect bacterial growth. One possible explanation is that disulfiram induces a cellular stress response, during which bacteria produce reactive oxygen species as part of a nonspecific redox-balancing mechanism. However, this ROS production may ultimately contribute to self-damage and growth inhibition. It is also possible that this is not an active detoxification strategy but rather an unintended consequence of disrupted redox homeostasis.

Disulfiram-treated *E. coli* upregulated many Fe–S clusters that bind or repair associated transcripts. Fe–S clusters are required in critical biological processes, including gene expression, respiration and metabolism (Roche et al., 2013). This metallocofactor annotates more than 100 proteins in *E. coli*, representing approximately 3% of the proteome (Bak and Weerapana, 2023). Moreover, Fe is associated with oxidative reactions because redox enzymes typically select Fe-based centers due to their high redox and oxygen sensitivity. Thus, Fe–S clusters could be sensitive to oxidation and may degrade under certain conditions (Chen et al., 2023). Zinc is a relatively redox-inert metal and is the most frequent substitute for the Fe–S cofactor site to maintain protein functions (Pritts and Michel, 2022; Imlay et al., 2019). However, during disulfiram treatment, zinc deprivation maintains the growth of *E. coli* or *S. aureus,* and zinc supplementation facilitates growth inhibition and ROS production. These results contrast with the previous “helping” role of zinc, indicating that zinc may be detrimental to bacteria under such oxidative conditions.

Zinc is associated with ROS production in some unknown ways. Recently, reports have shown correlations between zinc and oxidative status in biological systems (Arriaza et al., 2022; Liao et al., 2023); however, the link between these factors remains elusive. As a bivalent cation, Zinc does not change its oxidation state in cells. Thus, it may participate in redox reactions in an indirect manner. Metallothionein, a thiol-containing protein, is one of the zinc-binding and zinc-transferring proteins that may alter redox status when interacts with metals. However, whether these proteins induce bacterial ROS under such conditions remains to be explored. Although our data strongly support a zinc-dependent ROS-mediated pathway, the precise molecular interactions between disulfiram, zinc homeostasis, and ROS generation require further study.

One of the major limitations of disulfiram is that it is not a strong antimicrobial drug. Nevertheless, the mechanism of disulfiram warrants further exploration, as understanding the modes of action of chemical groups such as dithiocarboxy acids could inform the development of novel antibiotics.

## 5 Conclusion

This study identifies a zinc-dependent induction of intracellular ROS as a key mechanism underlying the antibacterial activity of disulfiram. The growth-inhibitory effect was mitigated by ROS scavengers and zinc chelators, highlighting the role of zinc and oxidative stress. These findings offer mechanistic insights into disulfiram’s antibacterial action and support its potential for therapeutic repurposing.

## Data Availability

The raw data supporting the conclusions of this article is available in Supplementary material. RNA-seq data was available with GEO accession numbers of GSE299097 and SRA accession numbers of PRJNA1272480 (https://www.ncbi.nlm.nih.gov/geo/query/acc.cgi?acc=GSE299097).
