# Supplementary material for: Disulfiram inhibits bacterial growth by inducing zinc-dependent reactive oxygen species
Source: Front Microbiol. 2025 Jul 17;16:1619416. doi: 10.3389/fmicb.2025.1619416 (PMC12310742; doi:10.3389/fmicb.2025.1619416)
Supplement: Supplementary file 1 [file Data_Sheet_1.PDF]

Supplementary Figure 1

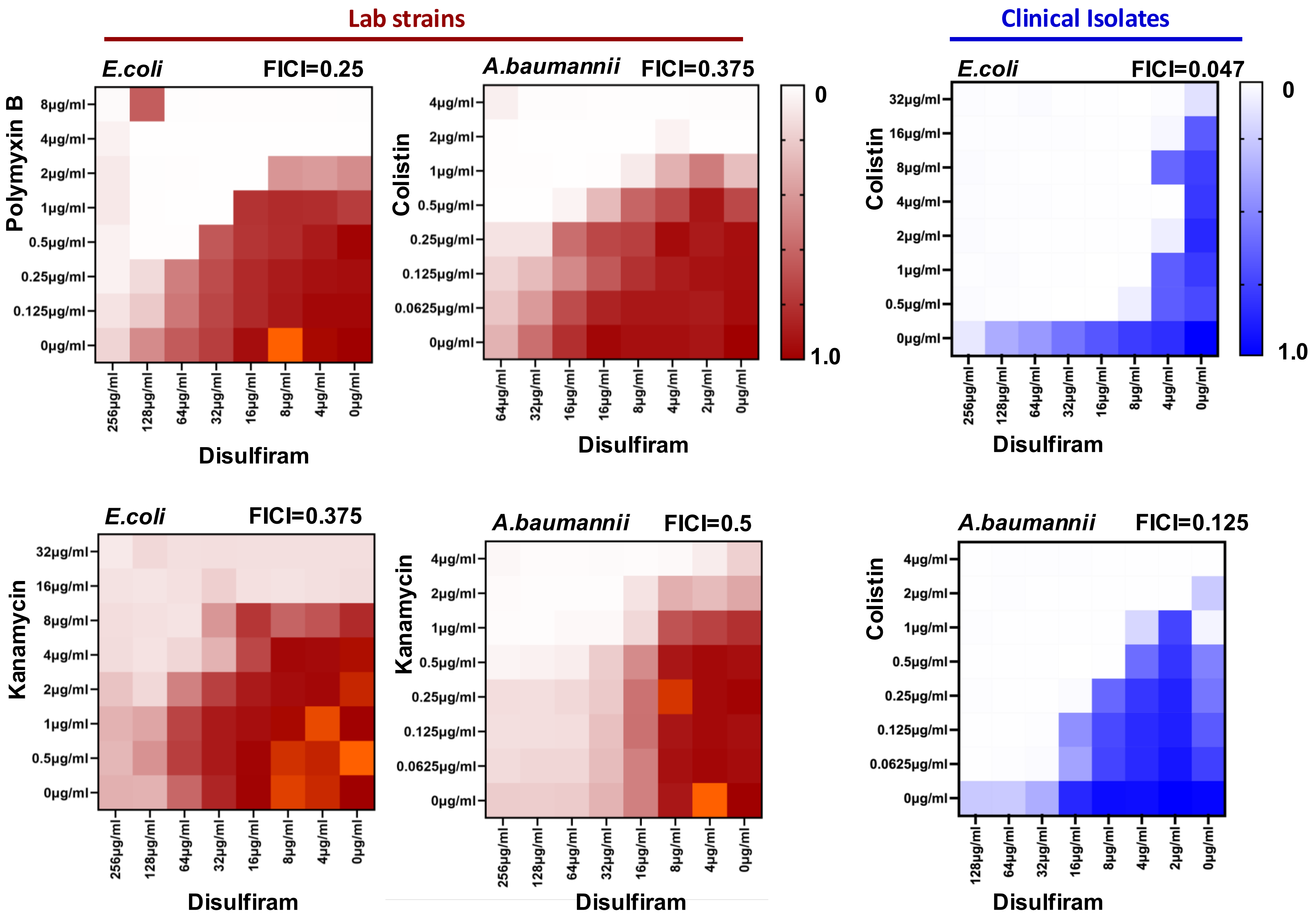

Supplementary Fig. 1| Checkerboard assay of lab strain or clinical isolated *E. coli* and *A. baumannii* treated with different drugs. The data represent the mean absorbance at 600 nm of biological replicates.

Supplementary Figure 2

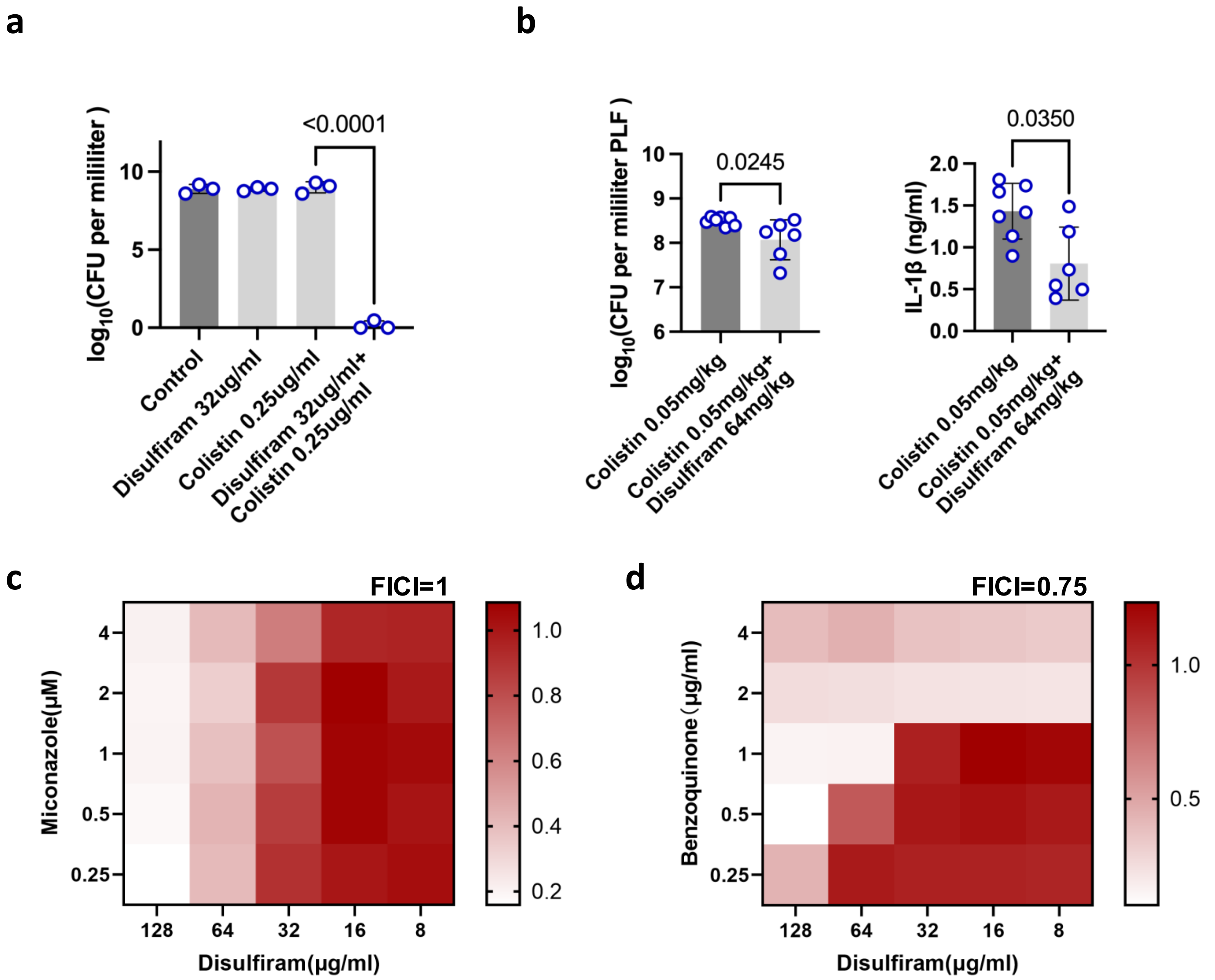

Supplementary Fig. 2| Flavohemoglobin may not be an efficient target to augment the effect of disulfiram. a, CFUs of *E. coli* in the presence of disufiram and colistin at 24 h. **b**, Two groups of mice (n = 6-7 per group) were intraperitoneally challenged with 7 × 10^6 CFU of *E. coli* and then administered a single-dose treatment as indicated. At 8 h posttreatment, samples were collected from peritoneal lavage fluids (PLF) for bacterial quantification (left) and IL-1β measurement (right) **c-d**, Checkerboard assay of *E. coli* treated with disulfiram and miconazole or benzoquinone. The data represent the mean absorbance at 600 nm of biological replicates. The means±SD are shown in **a** and **b**. P values were determined using one-way ANOVA (**a)** or student's *t* test (**b**).

Supplementary Figure 3

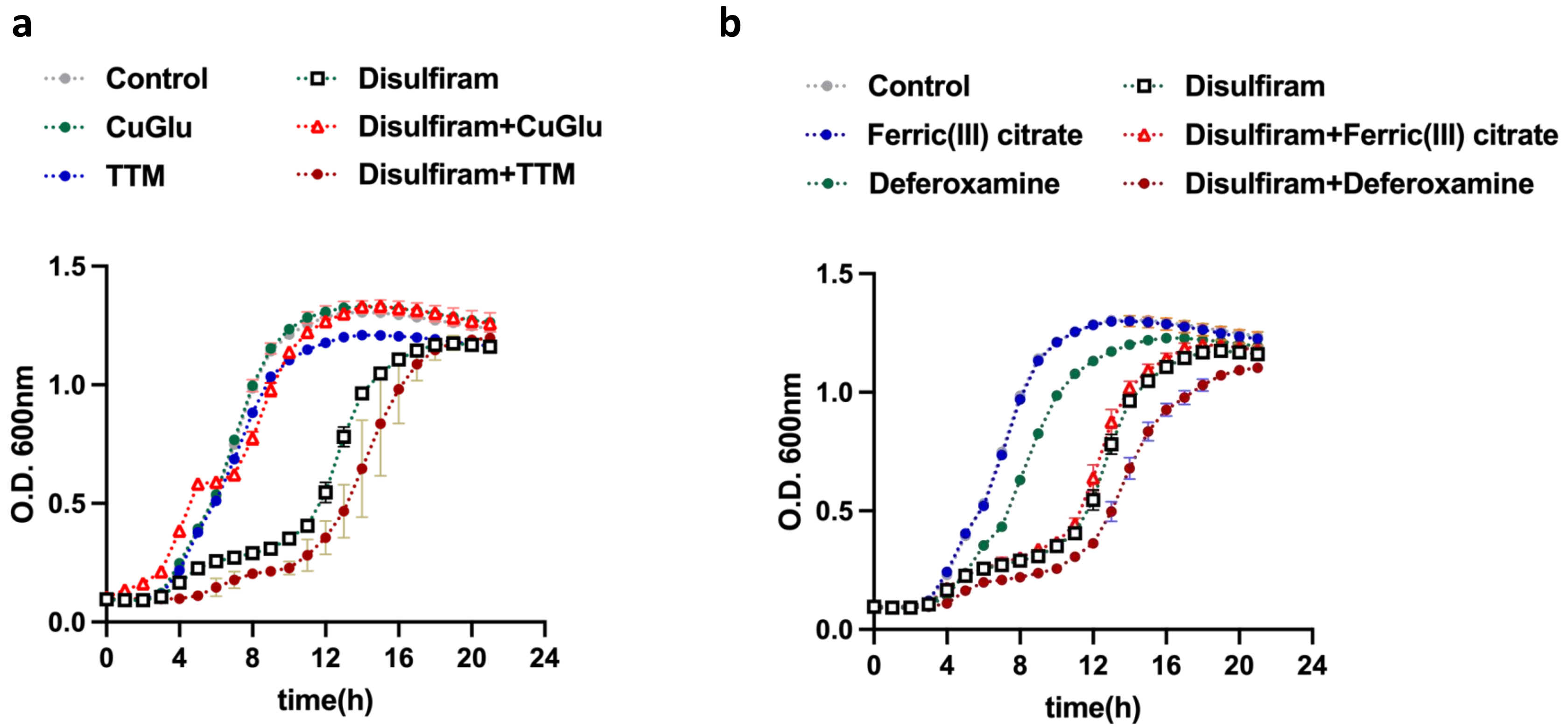

Supplementary Fig. 3| Effects of ferric or copper ions or their chelators on disulfiram inhibition of *E. coli*. a-b, Growth curves of *E. coli* treated with disulfiram (32 μg/ml), CuGlu (20 μM), TTM (20 μM), FAC (20 μM), DFO (20 μM) or their combination as indicated for 24 h. Data are the means±SD of three biological replicates.
